# Intestinal microbiota and arthritis

**DOI:** 10.1016/j.ero.2026.03.017

**Published:** 2026-05-08

**Authors:** Emilie Dumas, Dirk Elewaut

**Affiliations:** 1 VIB Center for Inflammation Research, VIB, Ghent, Belgium; 2 Department of Rheumatology, Ghent University Hospital, Ghent, Belgium

## Abstract

Intestinal dysbiosis appears as a common denominator among several rheumatic diseases. Here, we provide an overview of the most commonly occurring themes in the relationship between intestinal microbiota, host response, and arthritic disease.

Arthritis, eg, inflammation affecting one or more joints, is a leading cause of work disability worldwide. Although >100 distinct subtypes exist, osteoarthritis (OA) is the most prevalent, affecting an estimated 595 million individuals globally in 2020 [1]. OA is closely associated with ageing, impaired cartilage repair mechanisms, and chronic mechanical stress. Rheumatoid arthritis (RA), which impacted 17.6 million people worldwide in 2020 [2], represents one of the most common autoimmune disorders. Equally prevalent forms of inflammatory arthritis include spondyloarthritis, which can involve the axial skeleton and peripheral joints. Despite their diverse clinical presentations, a unifying feature among these musculoskeletal disorders is the emerging link between the gut microbiota and the onset, progression, and inflammatory burden of disease.

Barrier sites such as the gut, skin, and lungs function as dynamic immunological interfaces that regulate host exposure to environmental and microbial signals, rather than serving merely as static anatomical boundaries [3]. Compromise of these interfaces—termed barrier dysfunction—has emerged as a hallmark of multiple arthritic disorders [3], [4], [5]. The gastrointestinal tract, in particular, plays a central role in maintaining immune tolerance. The gut microbiota contributes to human health through nutrient metabolism, vitamin biosynthesis, maintenance of the intestinal epithelium, and modulation of both innate and adaptive immune responses [[6], [7]]. Disruptions in microbial community structure, collectively referred to as gut dysbiosis, have been observed across a wide spectrum of systemic inflammatory diseases, including those affecting the joints. Loss of barrier integrity has been reported in various contexts, such as the skin in psoriatic arthritis, the gut in spondyloarthritis, and the lungs and oral cavity in RA, highlighting the systemic nature of barrier-mediated immune perturbations. Here, we provide an overview of emerging themes in intestinal microbiota and arthritis.

## GUT MICROBIOTA DYSBIOSIS IN ARTHRITIS

Multiple studies have consistently demonstrated that arthritis is associated with reduced bacterial richness, often accompanied by depletion of beneficial short-chain fatty acid-producing bacteria and an enrichment of proinflammatory taxa [8]. Disease-specific bacterial patterns, however, remain somewhat heterogeneous between different types of arthritis. In RA, expansions of *Prevotella copri* and *Akkermansia* have been documented in several cohorts [[9], [10]], whereas in spondyloarthritis, associations have been noted with *Ruminococcus gnavus* [[11], [12]], genus *Dialister* [13], *Escherichia coli* [14]], and *Faecalibacterium prausnitzii* [15], occasionally correlating with disease activity. In OA, increased abundance of *Streptococcus* and *Enterococcus* has been linked to greater pain severity [[16], [17]]. Cross-study comparisons are complicated by methodological variability in biospecimen collection, DNA extraction, sequencing platforms, and analytical pipelines. Furthermore, patient-specific factors, particularly medication use, influence gastrointestinal ecology. Commonly prescribed agents such as nonsteroidal antiinflammatory drugs, corticosteroids, and disease-modifying antirheumatic drugs can alter barrier integrity, reduce short-chain fatty acid-producing taxa, or modify microbial metabolism [18], [19], [20], [21]. Reciprocally, the gut microbiota can influence response to medication by metabolising drugs and contribute to interpersonal variation in treatment efficacy and toxicity [22], [23], [24], [25].

Bioinformatic advances have highlighted that nonquantitative analytic approaches may obscure true microbial load changes, further complicating interpretation [26]. Despite compelling associations between gut dysbiosis and arthritis, definitive mechanistic links connecting microbial perturbations to joint pathology have yet to be convincingly determined.

## IS THE LINK BETWEEN GUT AND JOINT INFLAMMATION CAUSAL?

A prevailing hypothesis proposes a gut-joint axis, in which gut inflammation precedes and contributes to joint pathology. In this multistep model, epithelial inflammation drives gut dysbiosis and compromises mucosal barrier integrity, permitting the translocation of bacteria or microbial metabolites into the lamina propria. These signals activate innate immune cells, which traffic systemically and trigger inflammatory cascades in peripheral joints [27], [28]. Translocation from the intestinal lumen can increase infection risk via the systemic circulation and other organs, such as the liver, leading to metabolic disorders that also contribute to shaping the immune response [29]. Nevertheless, this framework does not fully account for patients exhibiting joint inflammation without clinically apparent gut disease, suggesting that gut and joint inflammation may reflect parallel, convergent immunological mechanisms rather than a strictly unidirectional process. As emphasised by Kuhn et al [30], attributing arthritis solely to a ‘leaky gut’ oversimplifies the complex immunopathology of these disorders. Gut inflammation, microbiota dysbiosis, and joint inflammation likely represent interconnected perturbations in immune regulation, each capable of amplifying the other.

Experimental evidence supports a role for microbial modulation in OA. Huang et al [31] demonstrated reduced severity of surgically induced OA in germ-free mice, whereas faecal microbiota transplantation (FMT) from a patient with metabolic syndrome exacerbated disease in colonised animals. Sophocleous et al [32] further reported that probiotic administration following healthy donor FMT mitigated cartilage damage in a destabilisation of the medial meniscus model [32], highlighting the capacity of the gut microbiota to modulate experimental OA.

In RA, interesting mechanistic hypotheses have been proposed, such as molecular mimicry, in which microbial antigens resemble joint-derived proteins. Another hypothesis suggests that bacteria may play a role in posttranslational modifications. Specifically, bacterial enzymes can modify both host and microbial proteins. For example, it has been demonstrated that peptidylarginine deiminases from *Porphyromonas gingivalis* contribute to citrullination. This implies that other bacterial enzymes or modified proteins might also trigger autoimmunity [33], [34], [35].

In this section, we highlighted the importance of reevaluating the gut-joint axis in rheumatology and recognising the potential for multiple immunological events to converge simultaneously, rather than occurring in a linear cause-and-effect manner.

## IS DYSBIOSIS LINKED TO THE ABILITY TO INDUCE REMISSION?

Predicting therapeutic response in rheumatology remains a challenge due to substantial interpatient variability. Clinicians currently lack reliable biomarkers to stratify patients according to likely responsiveness to specific drug classes. In oncology, gut microbiota composition has emerged as a strong determinant of immunotherapy success, with reduced microbial diversity linked to poor response to immune checkpoint inhibitors [36]. Although rheumatology research is more limited, one recent metagenomic study in treatment-naive, patients with new-onset RA has identified bacterial functional pathways—rather than individual taxa—associated with treatment resistance [37]. The reversibility of gut dysbiosis has spurred interest in microbiome-targeted interventions. Baseline microbial profiling may enable prediction of responsiveness to prebiotics, probiotics, live biotherapeutic products, and FMT. Although FMT has shown remarkable efficacy in *Clostridioides difficile* infection, its application in chronic immune-mediated disorders remains experimental. A recent trial in psoriatic arthritis patients receiving methotrexate identified 2 distinct gut microbiota profiles: a P-type dominated by *Prevotella* species and a B-type dominated by *Bacteroides* species, the latter exhibiting higher microbial richness. Patients with the B-type profile demonstrated improved clinical responses to FMT [38]. In RA, although some studies reported improvements in disease outcomes with prebiotic or probiotic interventions [39], [40], [41], others reported no significant change [42], [43]. These findings underscore the potential for microbiome-guided therapeutic strategies, although interindividual variability in microbial baseline composition, engraftment, and clinical outcomes highlights the need for further mechanistic studies. Future directions may include rational patient stratification, donor-recipient matching, adjunctive dietary or probiotic interventions, and engineered microbial consortia designed to restore immune-regulatory function.

In this section, we emphasised the significance of gut microbiota profiling in predicting how patients will respond to treatment. It is essential that baseline profiling serves as the foundation for patient stratification and the development of microbiota-driven therapies. These approaches should be tailored to each patient group rather than being solely based on clinical measures.

In sum, in this review, we provide an overview of some of the key questions linking intestinal microbiota and arthritis. We highlight potential future directions to bridge the gap between observational gut microbiota research, disease mechanisms, and treatment.

## CRediT authorship contribution statement

**Emilie Dumas:** Writing – review & editing, Writing – original draft. **Dirk Elewaut:** Writing – review & editing, Writing – original draft.

## Competing interests

DE has received financial support from the Vienna Medical Academy. DE reports a relationship with MRM Health NV that includes consulting or advisory services. ED declares no competing interests or personal relationships that could have appeared to influence the work reported in this paper.
